# Supplementary material for: Learning to see colours: Biologically relevant virtual staining for adipocyte cell images
Source: PLoS One. 2021 Oct 15;16(10):e0258546. doi: 10.1371/journal.pone.0258546 (PMC8519425; doi:10.1371/journal.pone.0258546)
Supplement: Attachment — Submitted filename: Responses to Reviewer.pdf [file pone.0258546.s001.pdf]

Responses to Reviewer

Dear reviewer,

Thank you for carefully going through our manuscript. Please find our responses to your comments below, shown in red text. In the revised manuscript we have made updates in line with your comments also using red text (and strikethrough of the old text where required).

Best regards, Philip J Harrison

PONE-D-21-15339

Learning to see colours: generating biologically relevant fluorescent labels from bright-field images

Review Comments to the Author

Reviewer #1: PONE-D-21-15339

In this manuscript, the authors apply the established approach of label-free microscopy to an adipocyte dataset with two . They modify previous deep-learning approaches using a variation on deep learning called Learning Using Privileged Information. They also use a feature-based approach to evaluate generated images, rather than the pixel-based approaches used in previous label-free work. There are a three significant issues with this manuscript that need to be addressed. First, relevant prior work is not cited (as itemized below). Second, the authors did not compare their approach to current state-of-the-art methods Without these comparisons it is unclear whether or not the manuscript contributes an advance to the field. Third, the images used in the study are not being made publicly available as required by journal policy.

Major issues:

1. The title, abstract and introduction are largely written as if the authors invented the approach of label-free microscopy, which is not correct. All should be modified to make clear the relationship of their work to prior work clear.

We did not intent to make it sound like we had invented label-free microscopy. We have added text in the abstract and references to additional articles (see response to your next comment) to deal with this concern.

2. The authors briefly mention Christiansen et al 2018 but do not mention the even more relevant papers from the Allen Institute for Cell Science (Johnson et al (2017) [https://arxiv.org/abs/1705.00092] and Ounkomol et al (2018) [https://doi.org/10.1038/s41592-018-0111-2]. They do not compare their approach to these state-of-the-art methods, which could been done either by applying the open source Allen Institute for Cell Science software to their images, or by applying their method to the publicly available images used by Ounkomol et al.

We agree that these two papers should have been cited in our manuscript. The Johnson et al paper we had not previously come across. Thank you for pointing out this paper to us, their method was interesting. We have added references to these two paper (see lines 66-72).

Concerning comparing our method to other state-of-the-art methods: As we tailored our methods to each imaging channel and our specific adipocyte image data, focusing on the derived features, and were not trying to devise a general method that would work for all virtual staining, we believe it would be difficult to devise a fair comparison. We can not directly apply these alternative methods directly to our dataset without making modifications to them. For instance, the Allen Institute for Cell Science software was developed for 3D reconstructions, not 2D as was our case. We have updated the text on line 102 to emphasize that we have build this method specifically for our adipocyte cell image data.

It is also the case that our method was actually compared against 7 other state-of-the-art methods in the competition organised by AstraZeneca and AI Sweden that we won. The 7 other teams used a variety of different and current approaches for the virtual staining. AstraZeneca and AISweden are in the process of writing a paper about the competition and the modelling solutions used by the competing teams. Unfortunately, this paper has not yet been completed nor published.

3. The idea of using features to evaluate synthetic images has been described previously by Zhao & Murphy (2007) [https://doi.org/10.1002/cyto.a.20487].

Thank you for bringing this paper to our attention, we have included a reference to it on lines 100-101.

4. While using features is appropriate, the authors should also calculate pixel-wise correlations in order to be able to compare their results to the previous work.

See previous response concerning comparing to other methods. We do however still report our pixel-level performance (MAEs) in Tables 2 and 3.

5. In order to enable others to reproduce and possibly improve upon the work and to comply with PLoS ONE policy, all images used in the study should be made publicly available. This reviewer strongly urges that the manuscript not be published without making the images freely available.

We would really like to make this data available, but unfortunately there are legal restrictions that make this impossible at the current time. At present access to the data can be requested from AI Sweden, although a sample of the data is freely available at https://www.ai.se/en/node/81535/adipocyte-cell-imaging-challenge.
