# Supplementary material for: Learning to see colours: Biologically relevant virtual staining for adipocyte cell images
Source: PLoS One. 2021 Oct 15;16(10):e0258546. doi: 10.1371/journal.pone.0258546 (PMC8519425; doi:10.1371/journal.pone.0258546)
Supplement: Attachment — Submitted filename: 2nd_responses_to_reviewer.pdf [file pone.0258546.s002.pdf]

Responses to Reviewer

Dear reviewer,

Thank you for going through our revised manuscript. Please find our responses to your comments below, shown in red text.

Best regards, Philip J Harrison

PONE-D-21-15339R1

Learning to see colours: generating biologically relevant fluorescent labels from bright-field images

PLOS ONE

Review Comments to the Author

Reviewer #1:

The authors have made a few welcome additions based upon my previous comments. They have decided not to compare their method to previous methods. While I understand their choice, in the absence of such a comparison the methodological significance of the work remains unknown. Therefore the work is best described as the application of an existing approach to a different cell type. I therefore strongly feel that the title is not appropriate and should be changed to something like “Development of label-free microscopy models for adipocytes”.

We agree, in light of your comments above, and those you made on the first draft of our manuscript that a less general title would be better. We have therefore changed the title of our manuscript to “Learning to see colours: biologically relevant virtual staining for adipocyte cell images”.
